# Analysis of molecular epidemiological characteristics and antimicrobial susceptibility of vancomycin-resistant and linezolid-resistant *Enterococcus* in China

**DOI:** 10.1186/s12920-024-01948-x

**Published:** 2024-07-01

**Authors:** Ping Pan, Long Sun, Xinyan Shi, Xian Huang, Yiping Yin, Beilei Pan, Lihua Hu, Qiang Shen

**Affiliations:** 1 Department of Clinical Laboratory, Hangzhou Women’s Hospital (Hangzhou Matenal and Child Health Care Hospital), Hangzhou, Zhejiang 310000 China; 2 Respiratory department, Zhejiang Provincial General Hospital of Chinese People’s Armed Police CN, Hangzhou, Zhejiang 310051 China; 3 Department of Hospital-acquired infection control, Zhejiang Provincial General Hospital of Chinese People’s Armed Police CN, Hangzhou, Zhejiang 310051 China; 4 Department of Critical Care Medicine, Zhejiang Provincial General Hospital of Chinese People’s Armed Police CN, Hangzhou, Zhejiang 310051 China; 5 Hangzhou xixi Hospital, Hangzhou, Zhejiang 310023 China

**Keywords:** Vancomycin, Linezolid, *Enterococcus faecalis*, *Enterococcus faecium*, Antibiotic resistance

## Abstract

**Background:**

This study investigates the distribution and characteristics of linezolid and vancomycin susceptibilities among *Enterococcus faecalis* (*E. faecalis*) and *Enterococcus faecium* (*E. faecium*) and explores the underlying resistance mechanisms.

**Methods:**

A total of 2842 *Enterococcus* clinical isolates from patients were retrospectively collected, and their clinical data were further analyzed. The minimum inhibitory concentrations (MICs) of vancomycin and linezolid were validated by broth dilution method. The resistance genes *optr*A, *cfr*, *van*A, *van*B and *van*M were investigated using polymerase chain reaction (PCR). Housekeeping genes and resistance genes were obtianed through whole-genome sequencing (WGS).

**Results:**

Of the 2842 *Enterococcus* isolates, 88.5% (2516) originated from urine, with *E. faecium* accounted for 60.1% of these. The *van*A gene was identified in 27/28 vancomycin resistant *Enterococcus* (VRE) isolates, 4 of which carried both *van*A and *van*M genes. The remaining strain was *van*M positive. The *optr*A gene was identified in all *E. faecalis* isolates among linezolid resistant *Enterococcus* (LRE). *E. faecium* showed a higher multiple antibiotic resistance index (MAR index) compared to *E. faecalis.* The multi-locus sequence typing (MLST) showed the sequence type of *E. faecium* mainly belongs to clonal complex (CC) 17, nearly *E. faecalis* isolates analyzed were differentiated into 7 characteristics of sequence types (STs), among which ST16 of CC16 were the major lineage.

**Conclusion:**

Urine was the primary source of VRE and LRE isolates in this study. *E. faecium* showed higher levels of resistance compared to *E. faecalis*. *Optr*A gene was detected in 91.6% of LRE, which could explain linezolid resistance, and *van* genes were detected in all vancomycin resistant *Enterococcus* strains, while *van*A was a key resistance mechanism in VRE identified in this study.

**Supplementary Information:**

The online version contains supplementary material available at 10.1186/s12920-024-01948-x.

## Introduction

*Enterococcus faecalis* (*E. faecalis*) and *Enterococcus faecium* (*E. faecium*) were major pathogens in healthcare-associated infections (HAIs), such as endocarditis, septicemia, urinary tract infections, and wound infections [1]. They possess a wealth of intrinsic resistance to cephalosporins, partial fluoroquinolones, aminoglycosides and so on. Meanwhile, *Enterococcus* isolates developed acquired resistance to β-lactam, aminoglycoside, tetracycline, erythromycin, chloramphenicol and rifampicin due to the widespread use of broad-spectrum antimicrobials [2]. In addition to intrinsic resistance and tolerance, enterococci have been extraordinarily successful at rapidly acquiring resistance to virtually any antimicrobial agent put into clinical use [3]. The plasticity of the *Enterococcus* genomes allowed them to rapidly respond and adapt to the environment by acquiring genetic determinants, which increased their ability to colonize and infect their host and cause diseases [4]. Meanwhile, the emergence of multidrug-resistant (MDR) *Enterococcus* had become a major public health threat (e.g. India, Japan) as it had limited the effective antimicrobial agents available to treat infections [5, 6], such as vancomycin resistant *Enterococcus* (VRE), linezolid resistant *Enterococcus* (LRE), and even the linezolid resistant vancomycin resistant *Enterococcus* (LRVRE), has increasingly challenged clinical treatments, as treatment options for VRE bacteremia were limited, the emergence of linezolid resistance as a result of selective pressure was of concern [7–10]. At present, *van* gene clusters were regarded as the most common mechanism of acquired vancomycin resistance [11], while *cfr*, *optr*A and mutation in 23s rRNA were recognized as prevalent mechanisms of linezolid resistance [12–14]. Recent studies have sought to establish a relationship between the phenotypic and genotypic drug resistance, or between the bacterial species and resistance genes in *Enterococcus* [15, 16], for instance, *van*A type had been characterized by acquired resistance to high levels of both vancomycin and teicoplanin, of which *van*A was mainly detected in vancomycin resistant *Enterococcus faecium* (VRE*fm*), *van*B type mediated resistance to vancomycin and had a broad MIC, but was sensitive to teicoplanin, *van*M-positive VRE showed heterogeneous resistance to vancomycin and teicoplanin [17, 18]. Therefore, it is very necessary for continuous surveillance and understanding of antimicrobial resistance mechanisms in *Enterococcus* species to guide appropriate therapeutic strategies. This paper briefly elucidates the distribution of specimens, drug susceptibility phenotypes and molecular characteristics of *E. faecium* and *E. faecalis* isolates from patients between 2012 and 2021.

## Materials and methods

### Bacterial isolates

2842 non-duplicated clinical isolates of *Enterococcus* (including *E. faecalis* and *E. faecium*) from hospital of Zhejiang people’s armed police between 2012 and 2021 were analyzed. There were 75 LRE and 39 VRE. However, only 28 VRE and 12 LRE stains were successfully revived for further study, of which were confirmed using matrix-assisted laser desorption/ionization time-of-flight mass spectrometry (MALDI-TOF MS) systems, Vitek MS (bioMerieux, France).

### Antibiotic susceptibility test

Antimicrobial susceptibility testing was conducted by BD PhoenixTM100 automatic microbial identification analyzer via BD Phoenix^™^ PMIC/ID-55. We used the broth dilution method to test the minimum inhibitory concentration (MIC) of 28 VRE to vancomycin and 12 LRE to linezolid according to the Clinical and Laboratory Standards Institute guidelines (CLSI M100 31th) [19], and considered vancomycin MIC: susceptible, ≤ 4 µg/mL; intermediate, 8–16 µg/mL; resistant, ≥ 32 µg/mL, However, linezolid MIC was considered as: susceptible, ≤ 2 µg/mL; intermediate, 4 µg/mL; resistant, ≥ 8 µg/mL. *E. faecalis* ATCC 29,212 was used as a quality control.

### Detection of Vancomycin and linezolid antimicrobial resistance genes

28 VRE and 12 LRE isolates were all verified to be consistent with the results of standard biochemical reactions. We took common resistance genes *van*A, *van*B, and *van*M gene for vancomycin resistance, and *cfr* and *optr*A for linezolid via polymerase chain reaction (PCR) [20]. The primers used in study were listed in Additional file 1.

### Retrospective whole genome sequencing and analysis

We performed whole genome sequencing (WGS) on 28 VRE and 12 LRE isolates. Genomic DNA was extracted using a QIAamp DNA Micro Kit (QIAGEN, 56,304). The library was sequenced on a Novaseq 6000 platform (Illumina Inc., San Diego, CA, USA) and 150 bp paired-end reads were generated use default parameters. The detail of WGS was described in Additional file 2.

### Statistical analysis

Using the WHONET 5.4 software, we conducted a statistical analysis total of 2842 *Enterococcus* strains in this study. Data analysis was performed using GraphPad Prism 8.0.2. Chi-square analysis was used to analyze the differences in the prevalence of the tested features in *E. faecalis* and *E. faecium* strains. The Mann Whitney test was used to analyze the amount of antibiotic resistance between linezolid resistant *Enterococcus faecalis* (LRE*fa*) and VRE*fm* (no statistical analysis was performed on vancomycin resistant *Enterococcus faecalis* (VRE*fa*) and linezolid resistant *Enterococcus faecium* (LRE*fm*) due to the presence of only one data point). *P* –value < 0.05 was considered statistically significant.

## Results

### Distribution of isolates

Over the past decade, 2842 *Enterococcus* isolates were detected. Among these, *E. faecium* (*n* = 1618, 56.9%) was more prevalent than *E. faecalis* (*n* = 1224, 43.1%). The majority of these Enterococci were isolated from urine specimens (2516/2842, 88.5%), followed by secretions (108/2842, 3.8%). Interestingly, *E. faecium* was found more frequently than *E. faecalis* in urine, pleural effusion, and ascites samples. However, in secretions, respiratory tract samples, pus, cerebrospinal (CSF), vaginal discharge, and blood, *E. faecalis* was detected in higher numbers than *E. faecium* (Table 1).

**Table 1:** Samples distribution of *Enterococcus* isolates from 2012 to 2021

Samples	E. faecalis		E. faecium		Total	
	Num.	Ratio (%)	Num.	Ratio (%)	Num.	Ratio (%)
Urine	1003	81.9	1513	93.5	2516	88.5
Secretions	82	6.7	26	1.6	108	3.8
Sputum	38	3.1	19	1.2	57	2.0
Blood	25	2.0	16	1.0	41	1.4
Pus	21	1.7	4	0.2	25	0.9
Throat swab	20	1.6	4	0.2	24	0.8
Cerebrospinal fluid (CSF)	8	0.7	5	0.3	13	0.5
Catheter	8	0.7	3	0.2	11	0.4
Bile	8	0.7	10	0.6	18	0.6
Vaginal discharge	7	0.6	1	0.1	8	0.3
Pleural effusion and ascites	4	0.3	17	1.1	21	0.7
Total	1224	100.0	1618	100.0	2842	100.0

**Fig. 1:**
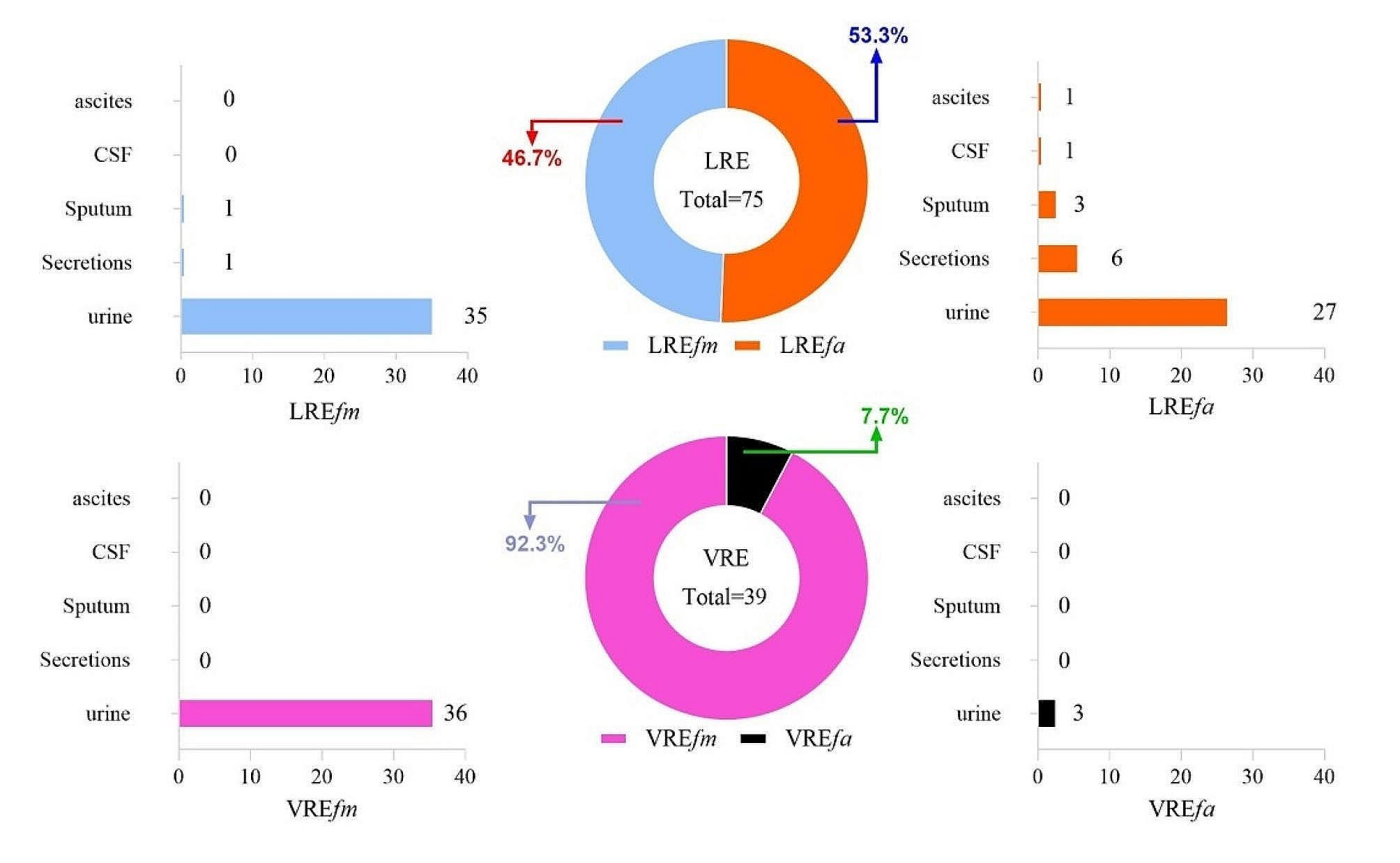
Characteristic of source distribution and strain composition between vancomycin resistant *Enterococcus* (VRE) and linezolid resistant *Enterococcus* (LRE). VRE and LRE were mainly detected in urine specimen, while the origin of LRE specimens was more extensive than VRE. VRE was dominated by *E. faecium*, whereas the detection of *E. faecalis* was comparable to *E. faecium* in LRE

A total of 39 VRE and 75 LRE isolates were further analyzed (Fig. 1; Table 2). This screening including 36 VRE*fm* and 3 VRE*fa* isolates, all of which were found in urine specimens. Additionally, the study included 38 LRE*fa* and 37 LRE*fm* isolates, which were recovered from urine, sputum, secretions, and CSF.

**Table 2:** Distribution of VRE and LRE

Samples	LREfa		LREfm		VREfm		VREfa	
	Num.	Ratio (%)	Num.	Ratio (%)	Num.	Ratio (%)	Num.	Ratio (%)
Urine	27	2.69	35	2.31	36	2.38	3	0.30
Others	11	4.98	2	1.90	0	/	0	/
Total	38	3.10	37	2.29	36	2.22	3	0.25

Note 1: Ratio of each strain in different samples was obtained by the following formula, urine sample as an example:

1 $$\text{R}\text{a}\text{t}\text{i}\text{o}\left(\text{\%}\right) \text{o}\text{f} \text{L}\text{R}\text{E}fs \text{i}\text{n} \text{u}\text{r}\text{i}\text{n}\text{e}=\frac{\text{T}\text{h}\text{e} \text{n}\text{u}\text{m}\text{b}\text{e}\text{r} \text{o}\text{f} \text{L}\text{R}\text{E}fs \text{i}\text{n} \text{u}\text{r}\text{i}\text{n}\text{e} }{\text{T}\text{h}\text{e} \text{n}\text{u}\text{m}\text{b}\text{e}\text{r} \text{o}\text{f} E. feacalis \text{i}\text{n} \text{u}\text{r}\text{i}\text{n}\text{e}}$$

Note 2: Others represent secretions, sputum, blood, pus, throat swab, cerebrospinal fluid, catheter, bile, vaginal discharge, pleural effusion, and ascites, etc.

Abbreviations: Num., number; VRE, vancomycin resistant *Enterococcus*; LRE, linezolid-resistant *Enterococcus* ; VRE*fm*, vancomycin resistant *Enterococcus faecium*; VRE*fa*, vancomycin resistant *Enterococcus faecalis*; LRE*fa*, linezolid resistant *Enterococcus faecalis*; LRE*fm*, linezolid resistant *Enterococcus faecium*; Num., number; LRE*fs*, linezolid resistant *Enterococcus*

### Antimicrobial susceptibility testing

The resistance rates of *E. faecalis* and *E. faecium* were shown in Additional file 3. In both sample types, *E. faecalis* exhibited the highest resistance to rifampicin (812/1003, 81%; 186/221, 84.1%). Conversely, *E. faecium* showed high resistance frequencies to ciprofloxacin (1465/1513, 96.8%; 97/105, 92.1%).

All 28 VRE strains showed 100% resistance to vancomycin, with high resistance observed (MIC ≥ 256 µg/mL). Meanwhile, 12 LRE strains were verified to have a linezolid resistance phenotype, showing variable resistance to linezolid (8 µg/mL to 16 µg/mL) (Additional file 4, Additional file 5).

### Multiple antibiotic resistance (MAR) index of VRE and LRE

The MAR (Multiple Antibiotic Resistance) index was used to analyse the resistance of 28 VRE and 12 LRE strains to 9 antibiotics (ampicillin, ciprofloxacin, rifampicin, penicillin, tetracycline, teicoplanin, vancomycin, nitrofurantoin, linezolid) using Eq. (1) in this study [21].

$$MAR\,index = \frac{{No.\,of\,anti\,biotics\,to\,which\,resistance\,occurred}}{{Total\,no.\,of\,antibiotics\,to\,which\,theisolatesweretested}}$$

**Fig. 2:**
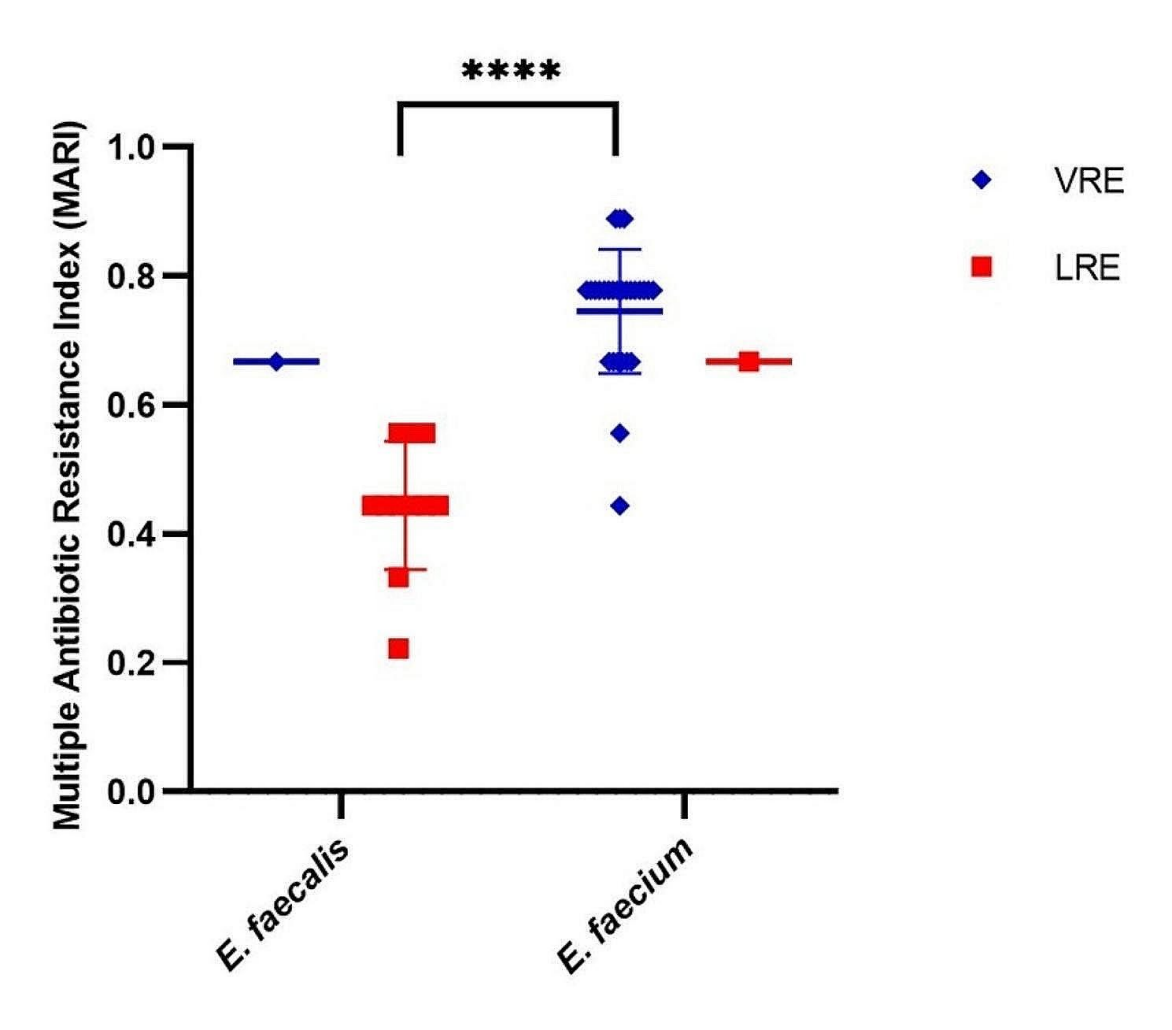
Multiple antibiotic resistance index (MAR index) of LRE and VRE. There was a significant difference between the median MAR index of LRE*fa* strains and VRE*fm* strains (*P*<0.0001)

It could be found that the MAR index of LRE strains ranged between 0.2 and 0.7, while VRE*fm* strains ranged between 0.4 and 0.9. VRE*fm* strains exhibited higher resistance to antibiotics than LRE*fa* strains (*P*<0.0001). All strains in this study were scored a MAR index of 0.3 or higher. Additionally, 19 out of 28 (67.9%) VRE strains were scored a MAR index of 0.8 or more. Therefore, we concluded that *E. faecium* showed extensive resistance to multiple evaluated antibacterial drugs and played larger MAR index values (Fig. 2, Additional file 6).

### The prevalence of drug resistance gene

*van* genes were detected in all VRE strains, and the prevalence of *van*A among VRE isolates was 96.4% (27/28), with 26 VRE*fm* and 1 VRE*fa*, while the remaining 1 VRE*fm* was found to carry a single resistance gene (*van*M), and among 26 VRE*fm*, 4 strains were found to carry 2 resistance genes (*van*A and *van*M). The prevalence of *optr*A among LRE isolates was 91.7% (11/12), all of which were detected in LRE*fa*, however, no resistance gene designed in this study was detected in a phenotypically positive strain. Unfortunately, *cfr* was not identified in the study (Additional file 4, Additional file 5).

### Characteristics of sequence types (STs)

In this study, multi-locus sequence typing (MLST) was performed for VRE and LRE based on the nucleotide sequences of seven housekeeping genes, respectively. Nine distinct STs were identified from 28 VRE isolates. The most prevalent type was ST78 (*n* = 17), which was prone to more variation in drug resistance gene profiles, followed by ST80 (*n* = 2), ST761 (*n* = 2), ST555 (*n* = 2). Additionally, there was only one strain each of ST262, ST789, ST6, ST17, and ST18. Meanwhile, seven STs were detected in 12 LRE strains, with ST1287 and ST16 dominating, each accounting for 25% (3/12). Additionally, ST256 (*n* = 1), ST409 (*n* = 1), ST480 (*n* = 1), ST911 (*n* = 1), and ST262 (*n* = 1) were also found in LRE strains (Additional file 4, Additional file 5).

## Discussion

In this study, we provided data on the characterization of clinical *Enterococcus* isolates including species, specimen distribution, prevalence of resistance genes, resistance phenotypes, and MLST among VRE and LRE.

In accordance with previous findings that *Enterococcus* spp. were frequently linked to urinary tract infections (UTIs) [22], the majority of *Enterococcus* in this study were isolated from urine (88.5%), predominantly, *E. faecium* (1618/2842, 56.9%). *E. faecium* had an inherent tenacity to develop resistance to antibiotics and environmental stressors, providing an advantage to thrive in hospital environments [23–25], thus, *E. faecium* showed a higher contribution than *E. faecalis* except in Pleural effusion and ascites.

According to the definition of enterococcal antibiotic resistance by Magiorakos et al., this study included 5/11 classes of antibiotics and 7/17 classes of antibiotics: Fluoroquinolones (Ciprofloxacin), Penicillins (Penicillin G, Ampicillin), Tetracycline, Glycopeptides (Vancomycin, Teicoplanin), and Oxazolidinones (Linezolid). All isolates (100%) were classified as extensive drug resistant (XDR) bacteria (defined as non-susceptibility to at least one agent in all but two or fewer antimicrobial categories) [26] (Additional file 6). Similar to our study, XDR strains were reported in 100% of *E. faecium* and *E. faecalis* in Egypt [27]. However, *E. faecium* was intrinsically more frequently reported as being more resistant to antibiotics, especially to vancomycin than *E. faecalis* [3]. And in our study, the detection of vancomycin resistance genes in 28 VRE strains also showed predominantly *van*-positive *E. faecium* (27 *van*-VRE*fm*, 1 *van*-VRE*fa*). Additionally, a MAR index of greater than 0.3 indicates that bacteria had already developed in an area in which antibiotics were often administered [21], in our study, the MAR index of 0.3 and more were recorded for the majority (97.5%) of LRE and VRE isolates. One isolate showed a 0.2 MAR index (LZD34), indicating certainly the link to heavy and uncontrolled use of antibiotics which might create a high antibiotic selective pressure in the hospital treatment. Therefore, the current isolates represent a high public health risk, which made many drugs unusable.

As mentioned above, linezolid was the only Food and Drug Administration (FDA)-approved antibiotic indicated for VRE infections [28–30]. Resistance mechanisms to linezolid include mutations in 23 S rRNA, alterations in ribosomal proteins (L3, L4, and L22), and acquisition of transferable resistance determinants such as *cfr*-like genes, *optr*A and *poxt*A, or *cfr* encoding 23 S rRNA methyltransferase [31, 32]. In this study, the *optr*A was the main resistant mechanisms to linezolid resistant *Enterococcus* (LRE). All *E. faecalis*, with MICs mainly at 8 µg/mL (7/11) and 16 µg/mL (4/11), compared to 0.5 to 2 µg/mL linezolid-sensitive strains, highlighting *optr*A’s significant role in resistance [33]. Notably, a linezolid resistant *E. faecalis* strain (MIC = 16 µg/mL) displayed no detectable resistance gene in our research, suggesting other resistance mechanisms.

MLST revealed nine STs among VRE strains and seven STs of LRE strains. As we know, ST78 has been reported to be the predominant ST in *van*A- and *van*M-type VRE*fm* strains in China [34, 35]. And ST16 was the most frequent ST among LRE isolates [36]. The majority of VRE*fm* underscore the prevalence reported previously [37], whereas the predominance of LRE*fa* suggests that *E. faecalis* may develop resistance to linezolid more readily than *E. faecium*. Clonal complex 17 (CC17) was a polyclonal group consisting of multiple STs, the epidemiology of enterococcal infections has been attributed to the increased ability of a genogroup of *E. faecium* related to human pathogen designated CC17 to colonize the gastrointestinal tract of humans, cause severe diseases [38]. ST78, ST17, ST18 and ST80 belonged to CC17 in this study (21/28), and there was one *van*M-type CC17, which explained the higher resistance level of *E. faecium* than *E. faecalis* in our study. Similar to our report, ST78 of CC17 *E. faecium* strains had been reported previously in hospitalized patients, while the majority ST type of CC17 in Rao’s study was ST80 [39, 40]. *E. faecalis* isolates analyzed were differentiated into nine STs, among which ST16 of CC16 were the major lineage, similar to Aung’s study in Northern Japan [6].

Continuous emergence of VRE, and their resistance to other antibiotics like daptomycin, linezolid, oritavancin, and the evolution of new resistance mechanisms pose a serious global health threat [41]. In this study, *E. faecalis* showed better susceptibility to ampicillin and nitrofurantoin, and vancomycin resistance in urine samples was primarily associated with *E. faecium*, making ampicillin and nitrofurantoin suitable for treating *E. faecalis* infection from urine in our hospital. In addition, linezolid still could be an option for treating VRE*fm*, as there was a bias towards *E. faecalis* (both in our study and from other studies) [28, 42]. In this study, VRE strains were all high-level vancomycin-resistance (≥ 256 µg/mL), and the potential for *van*M-positive *E. faecium* to develop vancomycin-resistance with prolonged treatment, enhanced surveillance for *van*M is advised [11, 28]. The detection of *optr*A could indicate linezolid resistance, highlighting the need for PCR-based molecular assays in clinical laboratories to facilitate rapid diagnostics due to the variability and time-consuming nature of conventional culture-based methods [43]. Besides, in our hospital, the majority of patients are convalescing, requiring a long-term hospital care, it is important to strengthen the clinical management of patients with VRE or LRE, education of healthcare workers, implementation and observation of hand-washing practices; active surveillance cultures (cultures at hospital admission, weekly cultures, cultures of high-risk patients) and the consequent prompt isolation of VRE or LRE-positive patients; and early isolation of high-risk patients [44].

## Conclusions

This study showed that *E. faecium*, particularly VRE strains were all obtained from urine, while the sources of LRE specimens were diverse. *E. faecium* showed higher levels of resistance owing to genetic characterization. *van*A and *van*M were currently detected in VRE, meanwhile *optr*A was found in LRE.

Vancomycin was the front-line agent for the treatment of ampicillin-resistant enterococcal infections or in patients with severe β-lactam allergies until the emergence and dissemination of VRE [43] and it was worth noting that some *van*M-carrying enterococci exhibit phenotypic susceptibility to vancomycin, and could revert to a vancomycin-resistant phenotype. The absence of phenotypically vancomycin sensitive strains for *van*M detection in our study might result in a decrease in the proportion of *van*M carrying *Enterococcus* strains. From Rao’s research, there was a significant association between virulence genes and antibiotics due to the presence of mobile resistance and virulence determinants on conjugative transposon [5], but our study was lacking the detection and analysis of virulence. Above all, the *van*M gene among vancomycin phenotypically sensitive strains and assess virulence.

Antibiotic resistance was a growing threat to human health, primarily driven by the overuse of antibiotics in clinical medicine. Clinically, drug resistance emerges after a series of antibiotic treatments, this trend was reflected not only in the increasing proportion of isolates resistant to multiple antibiotics, but also in the evolution of resistance to specific antibiotics.

In summary, the effective combination of clinical strain’s sources, species identification, and drug resistance genotyping is of great significance for targeted clinical treatment. This approach aids healthcare providers and patients to make reasonable treatment decisions at the bedside. Besides, we will explore novel therapeutic strategies for combating multidrug-resistant *Enterococcus* infections in future.

### Electronic supplementary material

Below is the link to the electronic supplementary material.

Supplementary Material 1

Supplementary Material 2

Supplementary Material 3

Supplementary Material 4

Supplementary Material 5

Supplementary Material 6

## Data Availability

Data is provided within the manuscript or supplementary information files.

## Abbreviations

CC: Clonal complex
CSF: Cerebrospinal fluid
CLSI: Clinical and Laboratory Standards Institute guidelines
E.: Faecium Enterococcus faecium
E.: Faecalis Enterococcus faecalis
HAIs: Healthcare-associated infections
LRE: Linezolid resistant Enterococcus
LREfa: Linezolid resistant Enterococcus faecalis
LREfm: Linezolid resistant Enterococcus faecium
LREfs: Linezolid resistant Enterococcus
LRVRE: Linezolid resistant vancomycin resistant Enterococcus
MAR: Multiple antibiotic resistance
MALDI-TOF: Matrix-assisted laser desorption/ionization time-of-flight
MICs: Minimum inhibitory concentrations
MLST: Multilocus sequence typing
MS: Mass spectrometry
Num.: Number
PCR: Polymerase chain reaction
STs: Characteristics of sequence types
UTIs: Urinary tract infections
VRE: Vancomycin resistant Enterococcus
VREfm: Vancomycin resistant Enterococcus faecium
VREfa: Vancomycin resistant Enterococcus faecalis
WGS: Whole-genome sequencing
XDR: Extensively drug-resistant
