# Supplementary material for: Analysis of molecular epidemiological characteristics and antimicrobial susceptibility of vancomycin-resistant and linezolid-resistant Enterococcus in China
Source: BMC Med Genomics. 2024 Jul 1;17:174. doi: 10.1186/s12920-024-01948-x (PMC11218351; doi:10.1186/s12920-024-01948-x)
Supplement: Supplementary file 1 — Supplementary Material 1 [file 12920_2024_1948_MOESM1_ESM.docx]

Table 1 Analysis of *E. faecalis* and *E. faecium* to antibiotic in urine samples from 2012 to 2021

| Antibiotic | *E. faecalis* （n=1003） | |  | *E. faecium*（n=1513） | | χ2 | *P* value |
| --- | --- | --- | --- | --- | --- | --- | --- |
|  | Resistance Num. | rates (%) |  | Resistance Num. | rates (%) |  |  |
| Ampicillin | 122 | 12.2 |  | 1378 | 91.1 | 1560.169 | ＜0.001 |
| Nitrofurantoin | 113 | 11.3 |  | 1251 | 82.7 | 1239.337 | 0 |
| Ciprofloxacin | 571 | 56.9 |  | 1465 | 96.8 | 621.928 | 0 |
| Rifampicin | 812 | 81 |  | 1259 | 83.2 | 2.107 | 0.15 |
| Linezolid | 27 | 2.7 |  | 35 | 2.3 | 0.36 | 0.15 |
| Tetracycline | 773 | 77.1 |  | 340 | 22.5 | 728.842 | 0 |
| Teicoplanin | 9 | 0.9 |  | 30 | 2 | 4.657 | 0.032 |
| Vancomycin | 3 | 0.3 |  | 36 | 2.4 | 17.104 | 0 |
| Penicillin G | 518 | 51.6 |  | 793 | 52.4 | 0.142 | 0.714 |

Note: *P*＜0.05 means difference was statistically significant.

Table 2 Analysis of *E. faecalis* and *E. faecium* to antibiotic in other specimens from 2012 to 2021

| Antibiotic | *E. faecalis* （n=221） | |  | *E. faecium*（n=105） | | χ2 | *P* value |
| --- | --- | --- | --- | --- | --- | --- | --- |
|  | Resistance Num. | rates (%) |  | Resistance Num. | rates (%) |  |  |
| Ampicillin | 12 | 5.4 |  | 92 | 87.6 | 221.331 | 0 |
| Nitrofurantoin | 9 | 3.9 |  | 79 | 75.3 | 182.928 | 0 |
| Ciprofloxacin | 75 | 34.1 |  | 97 | 92.1 | 97.552 | 0 |
| Rifampicin | 186 | 84.1 |  | 94 | 89.5 | 1.688 | 0.235 |
| Linezolid | 11 | 5 |  | 2 | 2 | 1.755 | 0.237 |
| Tetracycline | 161 | 72.9 |  | 24 | 23.3 | 72.482 | 0 |
| Teicoplanin | / | / |  | / | / | / | / |
| Vancomycin | / | / |  | / | / | / | / |
| Penicillin G | 124 | 55.9 |  | 67 | 63.6 | 1.74 | 0.229 |

Notes: Other specimens refer to secretions, sputum, blood, pus, throat swab, cerebrospinal fluid (CSF), catheter, bile, vaginal discharge, pleural effusion and ascites. *P*＜0.05 means difference was statistically significant.
