# Supplementary material for: Analysis of molecular epidemiological characteristics and antimicrobial susceptibility of vancomycin-resistant and linezolid-resistant Enterococcus in China
Source: BMC Med Genomics. 2024 Jul 1;17:174. doi: 10.1186/s12920-024-01948-x (PMC11218351; doi:10.1186/s12920-024-01948-x)
Supplement: Supplementary file 2 — Supplementary Material 2 [file 12920_2024_1948_MOESM2_ESM.doc]

Primers used in this study

| Primers |  | Amplicons (bp) |
| --- | --- | --- |
| *cfr*-F | AACAAAGAATTAGTCGATTTGAGGA | 831 |
| *cfr*-R | CTTCTACCTGCCCTTCGTTTG |
| *oprtA*-F | ATCAATGGTGGATGAAGTCCGT | 1426 |
| *oprtA*-R | GGAGTTACGATCGCCTTTTACTG |
| *vanA*-F | GCTCAGAGGAGCATGACGTATC | 789 |
| *vanA*-R | CCGTTTCCTGTATCCGTCCTC |
| *vanB*-F | CGATCCGCACTACATCGGAAT | 997 |
| *vanB*-R | ATGTGGCGTACTTGGCATCC |
| *vanM*-F | GTATCGGTAAAATCAGCGGCA | 912 |
| *vanM*-R | CATCATACGTGGATAACGGCT |
