# Supplementary material for: Analysis of molecular epidemiological characteristics and antimicrobial susceptibility of vancomycin-resistant and linezolid-resistant Enterococcus in China
Source: BMC Med Genomics. 2024 Jul 1;17:174. doi: 10.1186/s12920-024-01948-x (PMC11218351; doi:10.1186/s12920-024-01948-x)
Supplement: Supplementary file 3 — Supplementary Material 3 [file 12920_2024_1948_MOESM3_ESM.docx]

**The protocol of whole genome sequencing**

**Samples collection and** **DNA Extraction**

Genomic DNA was extracted using a QIAamp DNA Micro Kit (QIAGEN, 56304). DNA concentration was quantified using a NanoDrop™ 2000 (Thermo Scientific, Waltham, MA) spectrophotometer, and verified by agarose gel electrophoresis. The amount of DNA extracted > 50 ng was required for library preparation prior to using the next-generation sequencing.

**Genomic library preparation and DNA sequencing**

Libraries were prepared using the TruePrepTM DNA Library Prep Kit V2 for Illumina (Vazyme). Using a single "transposase" enzymatic reaction, sample DNA is simultaneously fragmented and tagged with adapters, an optimized, limited-cycle PCR protocol amplifies tagged DNA and adds sequencing indexes. Individual libraries were assessed on the QIAxcel Advanced Automatic nucleic acid analyzer and then were quantitated through qPCR by the use of KAPA SYBR FAST qPCR Kits. At last, the library was sequenced on an Illumina Novaseq 6000 platform (Illumina Inc., San Diego, CA, USA) and 150 bp paired-end reads were generated.

**Assembly**

Raw sequence data were processed through fastp (Version 0.23.2) and clean reads obtained by removing adapter and poly-N sequences, as well as low quality reads. The Trimmed reads are assembled using the de novo assembly (Primary assembly) tool called Unicycler.

**Genome Component prediction**

Prokka v1.12 software was used for gene prediction, RNAmmer-1.2 and tRNAscan-SE v1.3.1 software was used to predict the rRNA and tRNA contained in the genome.

**Gene function**

The Resistance Gene Identifier (RGI) 4.0.3 software was utilized to annotate resistance genes based on the Comprehensive Antibiotic Resistance Database (CARD).
